# Implementation of preemptive fluid strategy as a bundle to prevent fluid overload in children with acute respiratory distress syndrome and sepsis

**DOI:** 10.1186/s12887-018-1188-6

**Published:** 2018-06-26

**Authors:** Franco Díaz, María José Nuñez, Pablo Pino, Benjamín Erranz, Pablo Cruces

**Affiliations:** 1 Área de Cuidados Críticos, Hospital Padre Hurtado, Santiago, Chile; 2 Pediatric Intensive Care Unit, Clínica Alemana de Santiago, Santiago, Chile; 3 Facultad de Medicina Clínica Alemana Universidad del Desarrollo, Santiago, Chile; 4 Pediatric Intensive Care Unit, Hospital El Carmen de Maipú, Santiago, Chile; 5 Centro de Investigación de Medicina Veterinaria, Escuela de Medicina Veterinaria, Facultad de Ciencias de la Vida, Universidad Andrés Bello, Avda. Republica 217, Santiago, Chile

**Keywords:** Fluid overload, Pediatrics, Mechanical ventilation, Sepsis, PARDS

## Abstract

**Background:**

Fluid overload (FO) is associated with unfavorable outcomes in critically ill children. Clinicians are encouraged to avoid FO; however, strategies to avoid FO are not well-described in pediatrics. Our aim was to implement a bundle strategy to prevent FO in children with sepsis and pARDS and to compare the outcomes with a historical cohort.

**Methods:**

A quality improvement initiative, known as preemptive fluid strategy (PFS) was implemented to prevent early FO, in a 12-bed general PICU. Infants on mechanical ventilation (MV) fulfilling pARDS and sepsis criteria were prospectively recruited. For comparison, data from a historical cohort from 2015, with the same inclusion and exclusion criteria, was retrospectively reviewed. The PFS bundle consisted of 1. maintenance of intravenous fluids (MIVF) at 50% of requirements; 2. drug volume reduction; 3. dynamic monitoring of preload markers to determine the need for fluid bolus administration; 4. early use of diuretics; and 5. early initiation of enteral feeds. The historical cohort treatment, the standard fluid strategy (SFS), were based on physician preferences. Peak fluid overload (PFO) was the primary outcome. PFO was defined as the highest FO during the first 72 h. FO was calculated as (cumulative fluid input – cumulative output)/kg*100. Fluid input/output were registered every 12 h for 72 h.

**Results:**

Thirty-seven patients were included in the PFS group (54% male, 6 mo (IQR 2,11)) and 39 with SFS (64%male, 3 mo (IQR1,7)). PFO was lower in PFS (6.31% [IQR4.4–10]) compared to SFS (12% [IQR8.4–15.8]). FO was lower in PFS compared to CFS as early as 12 h after admission [2.4(1.4,3.7) v/s 4.3(1.5,5.5), *p* < 0.01] and maintained during the study. These differences were due to less fluid input (MIVF and fluid boluses). There were no differences in the renal function test. PRBC requirements were lower during the first 24 h in the PFS (5%) compared to SFS (28%, *p* < 0.05). MV duration was 81 h (58,98) in PFS and 118 h (85154) in SFS(p < 0.05). PICU LOS in PFS was 5 (4, 7) and in SFS was 8 (6, 10) days.

**Conclusion:**

Implementation of a bundle to prevent FO in children on MV with pARDS and sepsis resulted in less PFO. We observed a decrease in MV duration and PICU LOS. Future studies are needed to address if PFS might have a positive impact on health outcomes.

**Electronic supplementary material:**

The online version of this article (10.1186/s12887-018-1188-6) contains supplementary material, which is available to authorized users.

## Background

During the last two decades, intravenous fluid administration has been the cornerstone of treatment of children with hemodynamic instability. Restoration of circulating blood volume and perfusion to tissues is the primary end point of this therapy, decreasing mortality and morbidity of critically ill children with clinical signs of poor perfusion and shock [1]. Supported by guidelines and protocols, rapid administration of intravenous fluid is currently one of the most frequent interventions in critical care [2–5]. Intravenous (IV) fluid resuscitation may be lifesaving, but many studies have found that positive fluid balance is associated with negative outcomes in many clinical scenarios [6–15]. Critically ill patients are especially prone to positive fluid balance due to excessive fluid input (resuscitation fluids, maintenance intravenous fluid, continuous drug infusions, blood products and IV treatments), limited elimination of fluids (due to counterregulatory mechanisms such as antidiuretic hormone secretion) and due to capillary leakage in the interstitium, resulting in organ edema and dysfunction [16–19]. Detrimental effects are very pronounced in organs with a high density of capillaries, such as the lung, due to capillary leakage associated with a high hydrostatic pressure [16, 19–21]. Fluid overload has been associated with pulmonary dysfunction, hypoxia, longer duration of mechanical ventilation and ICU stay in children and adults on mechanical ventilation (MV) [6, 7, 13, 15, 20, 22–24]. In contrast, in the setting of acute respiratory distress syndrome (ARDS), diuretic use and restrictive fluid management are associated with lower mortality and faster liberation from MV, respectively [18, 24–26].

Despite these overwhelming data, few studies in pediatrics have addressed how to avoid significant fluid overload in patients under MV.

The objective of this study was to describe the implementation of a preemptive fluid strategy in children with sepsis and ARDS. In addition, a comparison with an historical cohort of infants was made.

## Methods

Expedited review by the IRB approved the quality improvement project, waiving the requirement for a written consent.

Setting: During a 12-month period (January to December 2016), all children admitted to the Pediatric Intensive Care Unit at Hospital Padre Hurtado were screened. Our unit is a 12-bed general medical and surgical PICU and does not take care of patients after congenital heart surgery or those receiving a transplant.

Participants: All patients younger than 24-months who received MV and fulfilled the pediatric ARDS (pARDS) and sepsis criteria, were prospectively identified. pARDS was defined according to the PALICC criteria [27] and sepsis was defined according to the International Pediatric Sepsis Consensus Conference definitions [28]. Patients were excluded if they were younger than 28 days old, had chronic renal insufficiency or end-stage renal disease, required dialysis, had a cyanotic congenital heart disease, underwent tracheostomy or required the chronic use of a positive pressure ventilation system.

### Preemptive fluid strategy (PFS)

As previously reported [29], this protocol was developed following 5 principles, in the post-resuscitation phase of critical illness:

1. Maintenance of intravenous fluids (MIVF) at 50% of baseline requirements estimated by the Holliday-Segar formula, ensuring adequate glucose infusion rate for normoglycemia.
2. Preparation of continuous infusions of medications (sedation and vasoactive drugs) and intravenous treatments (e.g., antibiotics) concentrated to the minimum volume recommended. Use of miniaturized devices for hemodynamic monitoring (i.e., pressure transducers) at the minimum rate of saline infusion.
3. Use of dynamic preload markers (pulse pressure variation) in addition to clinical assessment to decide administration of fluid boluses and early titration of vasoactive drugs.
4. Consideration of early use of diuretics when hypovolemia was ruled out, resuscitation goals were met and urinary output was less than 0.5 ml/kg/h.
5. Early initiation of enteral feeds.

A Historical cohort data was collected retrospectively with the same inclusion/exclusion criteria from June 2014 to December 2015. These patients were treated with standard fluid strategy and compared with the prospectively collected data of the patients managed with PFS.

### Standard fluid strategy (SFS)

Standard fluid strategy can be summarized as:

1. Resuscitation phase: hemodynamic resuscitation based on Surviving Sepsis Campaign [2, 3].
2. Stabilization phase: patients received MIVF at 100% of baseline requirements estimated by the Holliday-Segar formula.
3. Depletive phase: Initiation of diuretics after meeting resuscitation end points, hemodynamic stability and absence of any marker of dysoxia.

### Data collection

Demographic and clinical data were recorded in a relational database. Fluid intake and output (I/O) were recorded every 12 h during the first 3 days after admission. To determine the causes of fluid overload and variations in clinical practice after initiation of the protocol, fluid intake was divided into MIVF, fluid bolus, enteral feeds, drug administration (e.g., antibiotics) and packed red blood cells (PRBC) transfusions. Renal function was monitored daily and electrolyte alterations were monitored every 12 h or more frequently depending on the treating physician’s assessment. Urinary output and diuretic use were also recorded.

Vasoactive drug (VAD) support was recorded and maximum vasoactive inotropic score (VIS) score was calculated daily [30]. The percent of fluid overload (FO) was calculated using the following formula: [(total fluid intake (L) − total fluid output in liters (L)) / (admission weight in kilograms)*100] [31]. Peak FO was defined as the maximum percentage of FO during the first 72 h after initiation of invasive MV. No data were collected before PICU admission.

The primary outcome was to measure peak fluid overload (PFO). Fluid input and output were recorded as secondary outcomes to determine the causes of FO. Other clinical outcomes such as duration of MV, hospital and PICU length of stay (LOS) were recorded.

### Statistical considerations

Descriptive statistics were used to summarize all continuous and categorical variables. Comparisons between patient groups were performed using Fisher’s exact test for categorical variables and the Mann-Whitney U test for continuous variables because of concerns about the normality of the distribution of these variables. Two-way repeated measures ANOVA test was performed for comparisons (fluid overload) among and between groups during the study period. All statistical tests were 2-sided and were performed with a *P*-value less than 0.05 indicating statistical significance. The SPSS software package (version 20.0; SPSS, Chicago, IL, USA) was used for the statistical analyses.

## Results

Thirty-seven patients were included in the PFS group and compared with 39 patients in the SFS group (Table 1). No mortality was noted in this cohort.

**Table 1:** Demographics and clinical outcomes of septic children with ARDS on preemptive fluid strategy and standard fluid strategy

	Preemptive	Conservative
N	37	39
Male	54%	64%
Age (mo)	6 (2,11)*	3 (1,7)
Weight (kg)	8.4 (5.4,10)*	5 (4.7,9)
Comorbility	38%	49%
RSV	54%	51%
P/F ratio at admission	207 (146,249)	193 (146,234)
Duration of MV (h)	81 (58,98)*	118 (85.5154.5)
PICU LOS (days)	5 (4,7)*	8 (6,10)

(*P < 0.05)

Abbreviations: *ARDS* acute respiratory distress syndrome, *RSV* respiratory syncytial virus; *P/F ratio* PaO_2_/FiO_2_ ratio, *MV* mechanical ventilation, *PICU LOS* pediatric intensive care unit length of stay

**Fig. 1:**
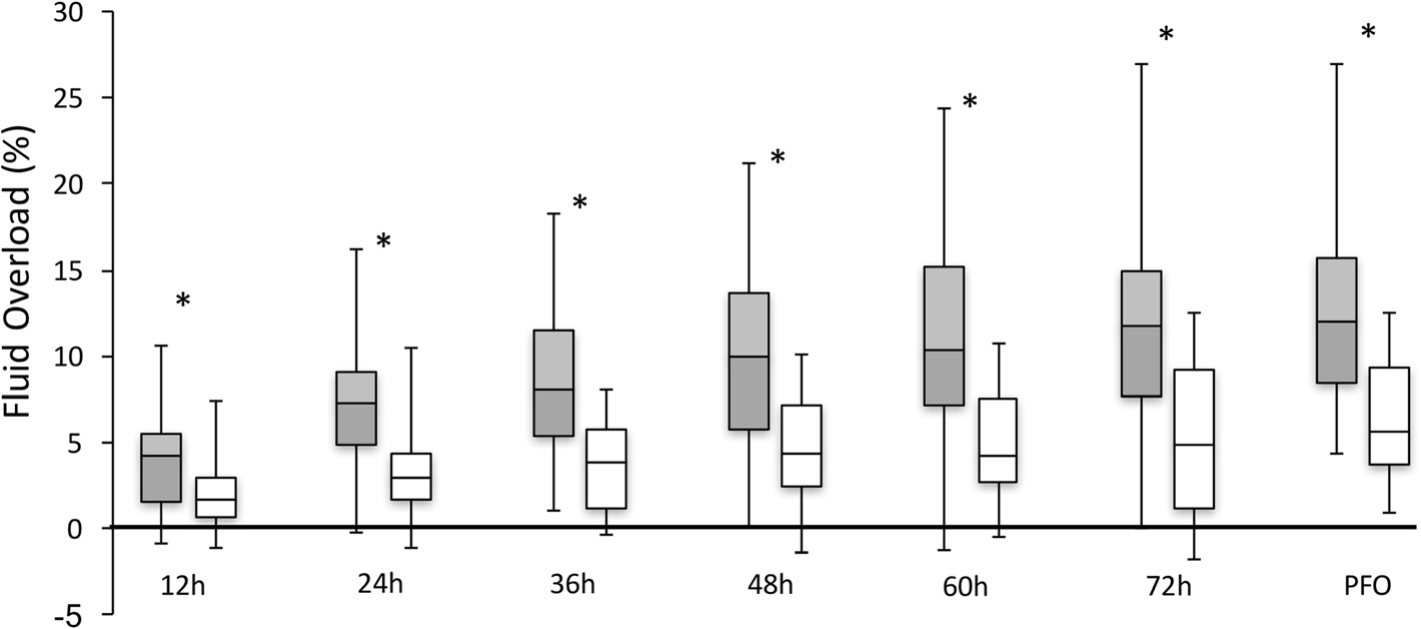
Box plot graph of cumulative and peak fluid overload during the first 72 h after admission in children with sepsis and ARDS with preemptive fluid strategy (white) and standard fluid strategy (gray). (* *P* < 0.05). Abbreviations: ARDS: acute respiratory distress syndrome; PFO: peak fluid overload

Fluid overload was significantly lower in the PFS group compared to the SFS group at 12, 24, 36, 48, 60 and 72 h (*P* < 0.05) (Fig. 1). PFO was significantly lower in the PFS group (6.31% [IQR 4.4–10]) compared to the SFS group (12% [IQR 8.4–15.8]).

**Fig. 2:**
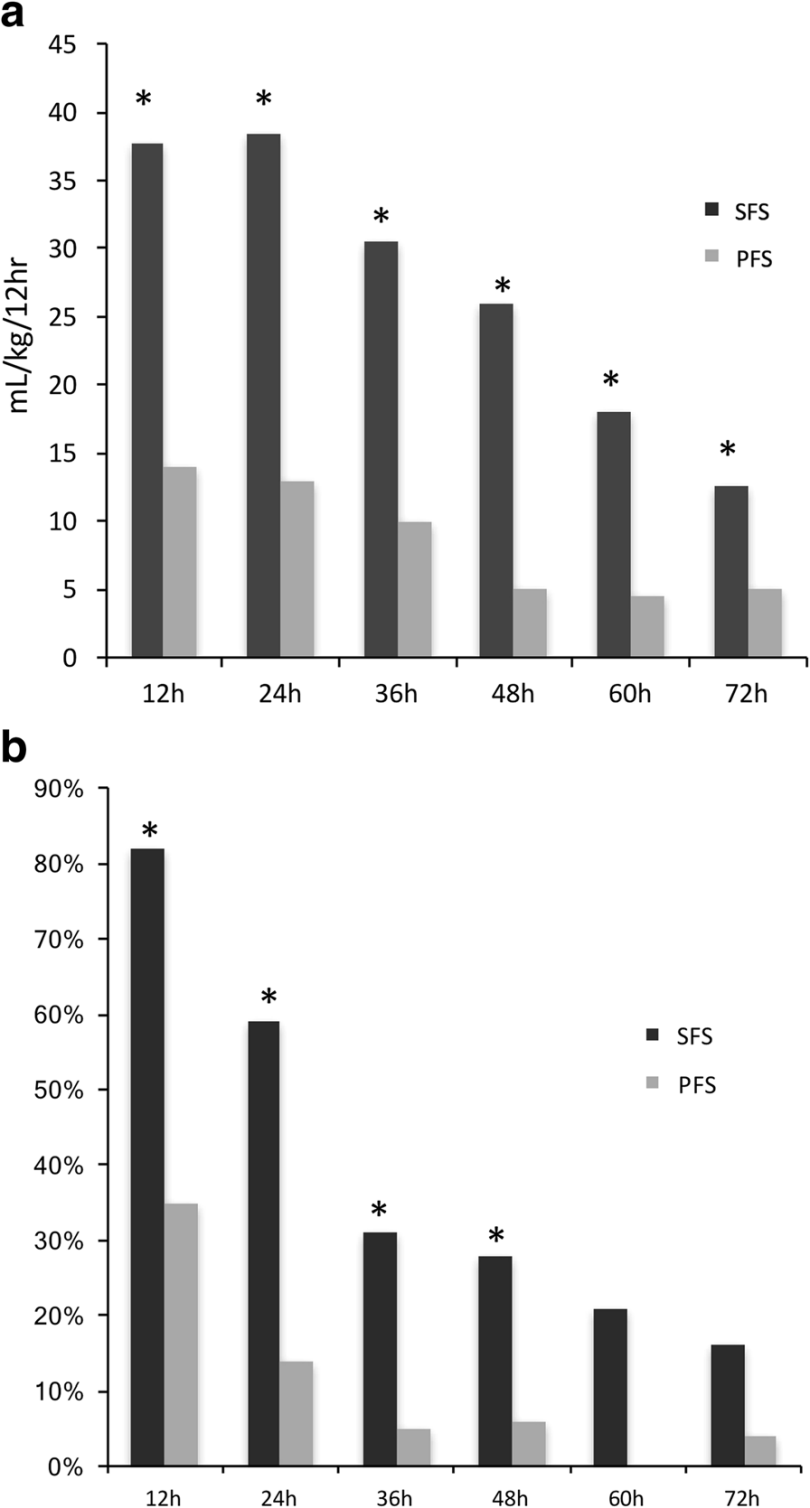
Maintenance intravenous fluid administration (ml·kg^−1^·12 h^−1^) in both groups during the study intervals after admission (**a**). Percentage of patients that received at least one fluid bolus during the study intervals after admission (**b**). (* P < 0.05). Abbreviations: PFS: preemptive fluid strategy; SFS: standard fluid strategy

The PFS group required significantly less fluid input due to less MIVF and fewer resuscitation boluses compared to SFS (Fig. 2a and b). The PFS group had a lower urinary output, but renal function tests were not different from the SFS group. Diuretic use was frequent in both groups, at 24 h 54% of patients on PFS received at least one diuretic bolus and 15% of patients on SFS (*P* = 0.003). The SFS group required more frequent continuous infusions of diuretics at 48 and 72 h. (Table 2).

**Table 2:** Renal function test, urinary output and percentage of patients that received diuretics in the preemptive fluid strategy and standard fluid strategy groups

	24 h		48 h		72 h	
	PFS	SFS	PFS	SFS	PFS	SFS
N	37	39	36	39	24	37
BUN (mg·dL^−1^)	7 (6,9.5)	4 (3,8)	5 (3,8)	3 (2,6)	7 (5,16)	4 (2,8.5)
Creatinine (mg·dL^−1^)	0.22 (0.16,0.28)	0.18 (0.14,0.24)	0.21 (0.19,0.3)	0.2 (0.15,0.26)	0.22 (0.14,0.35)	0.22 (0.19,0.36)
Urinary output (mL·kg^−1^·h^−1^)	1.8* (1.1,2.8)	2.9 (1.8,3.7)	2.3* (1.6,3.5)	4 (2.9,5.7)	2.6* (2.1,3.75)	4.1 (3,5.2)
Diuretic bolus (%)	54*	15	72	64	57	76
Continuous infusion of diuretic (%)	11	13	19*	59	17*	70

(* P < 0.05)

Abbreviations: *PFS* preemptive fluid strategy, *SFS* standard fluid strategy

PRBC transfusions were less frequent in the first 12 h after admission in the PFS group. Enteral feeds were started earlier in the PFS group (Table 3). The median duration of vasoactive drug support was 2 days (0,3) in the PFS group and 3 days [2, 4] in the SFS group (*P* = 0.018) and VIS was significantly lower during the first and the third day of admission in the PFS group. Use of greater than or equal to 2 vasoactive drugs was more frequent in the SFS group during the third day after admission (Table 3). Epinephrine support was more frequent in the SFS group during the first day after admission, whereas norepinephrine was more frequent during the third day after admission in the same group (Additional file 1: Figure S1).

**Table 3:** Percentage of patients of each group that received packed red blood cell transfusion, enteral feeds and 2 or more vasoactive drugs at different study points

	12 h		24 h		36 h		48 h		60 h		72 h	
	PFS	SFS	PFS	SFS	PFS	SFS	PFS	SFS	PFS	SFS	PFS	SFS
(N)	37	39	37	39	37	39	36	39	33	38	24	37
PRBC	5%*	28%	27%	26%	14%	10%	6%	10%	3%	5%	0%	3%
Enteral Feeds	16%*	0%	59%*	28%	84%*	49%	92%	77%	84%	87%	83%	78%
VIS			3.43	7.76*			5.07	7.87			3.22*	6.37
≥2 VAD			11%	18%			16%	31%			3%*	21%

Highest calculated vasoactive inotropic score during the first, second and third day. (*P < 0.05)

Abbreviations: *PFS* preemptive fluid strategy, *SFS* standard fluid strategy, *PRBC* packed red blood cell transfusion, *VAD* vasoactive drugs, *VIS* vasoactive inotropic score

Time to successful extubation was 81.5 h [IQR 56.5–100.5] in the PFS group and 118 h [IQR 85.5–154.5] in the SFS group (*P* < 0.01 by the log rank test). Additionally, PICU LOS was shorter in the PFS group compared to the SFS group (5 days [IQR 4–7] vs 8 days [IQR 6–10], *P* < 0.01).

## Discussion

The main finding of our study is that prevention of fluid overload as a bundle for critically ill children was successfully implemented in a general PICU. Critically ill children with sepsis and pARDS that underwent preemptive fluid strategy had less peak fluid overload compared with standard fluid strategy. These differences were due to a lower requirement for fluid input from MIVF and fluid bolus resuscitation, especially during the first 48 h. No detrimental effects were found on renal function test, urinary output or vasoactive drugs requirements.

The detrimental effects of fluid overload in critically ill children have been extensively reported in many different settings [6–15, 20–23], but this study is the first one to offer an alternative approach to avoid early fluid overload. We developed this bundle based on preliminary data (29) that showed that excessive fluid administration during the first 72 h after admission was the main responsible factor for early fluid overload during the course of critical illness.

Maintenance intravenous fluid calculation in current practices is based on the Holliday-Segar method, which was developed many decades ago. It is important to note that this method was supposed to estimate the 24 h water loss in hospitalized euvolemic children with normal renal function [32]. This method was not developed for use in critically ill infants, so it does not account for all the peculiarities of this group of patients. For example, febrile illness and tachypnea can increase insensible water loss. On the other hand, energy expenditure is lower in infants sedated and on MV and insensible water losses are very low in normothermic infants breathing humidified gas [32, 33]. Based on these data, we decreased MIVF to 50% of the calculated rate while maintaining the glucose infusion rates for infants to avoid hypoglycemia.

The second element of the bundle was fluid bolus administration. In addition to the clinical assessment of hypovolemia, we added to our bundle a dynamic preload parameter, pulse pressure variation, to decide on fluid administration. Resuscitation protocols and guidelines propose an aggressive fluid management approach as the first line treatment for sepsis and shock [1, 2]. The main objective of fluid bolus administration is to correct hypovolemia, a common initial finding in pediatric sepsis and shock [34]. Currently, fluid bolus administration is a frequent intervention in the emergency department and most of the patients receive between 20 and 60 mL·kg^− 1^ before PICU admission [35]. The risks and unwanted effects of this approach have been highlighted in recent studies [36–38]. Adequate fluid resuscitation increases venous return, end diastolic and systolic volume and consequently stroke volume and cardiac index. Despite the widespread clinical use, data supporting fluid bolus therapy in hospitalized critically ill children is very weak [39]. Recent adult studies have emphasized that liberal use of fluid bolus is associated with a positive fluid balance, exposing patients to the risks associated with fluid overload [24–26]. We acknowledge that the functional hemodynamic markers of preload are not widely used in critically ill children. Most devices providing invasive arterial pressure monitoring can give at least pulse pressure variation parameters with the current technology. In our view, functional hemodynamic monitoring gives to the clinician an additional parameter to assess fluid status and predict fluid responsiveness, along with vital signs, physical examinations and biomarkers of dysoxia. Only 50% of critical ill children are fluid responders [40], due to myocardial dysfunction and blunted adrenergic sensitivity [40, 41]. Additionally, it is important to recall that fluid hemodynamic response is short. It is estimated that 85% of crystalloid fluid boluses redistribute in the interstitial tissue four hours after administration or even less in critically ill patients having an increased capillary leak [5, 42]. In daily practice fluid boluses are the first response to multiple scenarios without a strong physiological support, i.e., tachycardia due to fever, respiratory distress or pain. With PFS, we were able to decrease fluid boluses, especially during the first 24 h, without any clinical evidence of detrimental hypovolemia or significant renal dysfunction. Urinary output was lower in PFS, and diuretic initiation was more frequent during the first 24 h after admission, but renal function tests where similar between groups. Limiting unnecessary fluid administration in the PFS group was also associated with less PRBC transfusions, probably as a result of a decreased hemodilution. Vasoactive drug support was lower in the PFS group. This may seem counterintuitive, but studies in adults have shown that hemodynamic instability is associated with liberal use of fluids [43–47].

The initiation of enteral feeds occurred earlier in the PFS group. From the practical standpoint, enteral feeds allow to decrease unnecessary MIVF, improve nutritional support and are essential for the metabolic homeostasis of critically ill patient. In addition, several non-nutritive benefits of enteral feeds have been described, such as anti-inflammatory and immunomodulatory effects, which may have a major impact on the outcome of patients with ARDS [48–50]. In our center, as in many PICUs, early initiation of enteral feeds has become a priority and a quality standard.

We found that PFS was associated with less MV days and PICU LOS, with similar findings being reported from studies in adults. Although this study was not designed to address these outcomes, we believe that these observations set the target and basis for future prospective studies in the field. A recent systematic review and meta-analysis reported that in adults and children with ARDS and sepsis, a conservative fluid strategy resulted in an increased number of ventilator-free days and a decreased length of ICU stay compared with a more liberal strategy or standard care [26].

Our study has some limitations. No data were gathered before PICU admission. The described strategy, PFS, does not apply for patients during resuscitation from septic shock. Adequate initial fluid resuscitation of patients in septic shock decreases mortality and has been well documented in many studies [1–4, 34], but the goals and metrics of these interventions need to be redefined. No specific data of the endpoints of resuscitation were registered, so it is difficult to extrapolate our protocol to unstable patients with ongoing signs of poor perfusion or dysoxia. Specifically, it is controversial what objective data the clinician must consider to terminate the resuscitation phase. All the patients included in this study suffered from sepsis due to acute pulmonary infectious diseases. Therefore, the findings in this study cannot be directly extrapolated to patients with extrapulmonary pARDS. Our measurements and outcomes were short-term and obtained upon PICU admission, so we cannot extrapolate the long-term effects using this approach. Finally, we must acknowledge the limitations of comparing our findings with a historical retrospective cohort, especially since selection bias and type II error cannot be ruled out.

## Conclusion

We successfully implemented a quality improvement initiative to prevent early fluid overload in critically ill children. In our view, clinicians must be aware that a systematic approach with measures specifically limiting unnecessary fluid input (MIVF and resuscitation fluid bolus) over the first 48 h can be easily applied in most PICU and may prevent significant FO.

Future collaborative studies are needed to address if a preemptive fluid strategy after resuscitation from shock, in addition to optimal care for pARDS (protective mechanical ventilation, avoidance of patient ventilator asynchrony, optimal nutrition, weaning protocol, among others) might have a positive impact in outcomes.

## Additional file

Additional file 1: **Figure S1.** Vasoactive drug use in standard fluid strategy and preemptive fluid strategy at day 1, 2 and 3 of study. * *P* < 0.05. Abbreviations: PFS: preemptive fluid strategy; SFS: standard fluid strategy. (JPG 226 kb)

## Abbreviations

ARDS: Acute respiratory distress syndrome
FO: Fluid overload
ICU: Intensive care unit
IV: Intravenous
MIVF: Maintenance intravenous fluids
MV: Mechanical ventilation
pARDS: pediatric acute respiratory distress syndrome
PFO: Peak fluid overload
PFS: Preemptive fluid strategy
PRBC: Packed red blood cells
SFS: Standard fluid strategy
VAD: Vasoactive drugs
VIS: Vasoactive/inotropic score
