# Supplementary figures and images for: Implementation of preemptive fluid strategy as a bundle to prevent fluid overload in children with acute respiratory distress syndrome and sepsis
Source: BMC Pediatr. 2018 Jun 26;18:207. doi: 10.1186/s12887-018-1188-6 (PMC6020419; doi:10.1186/s12887-018-1188-6)

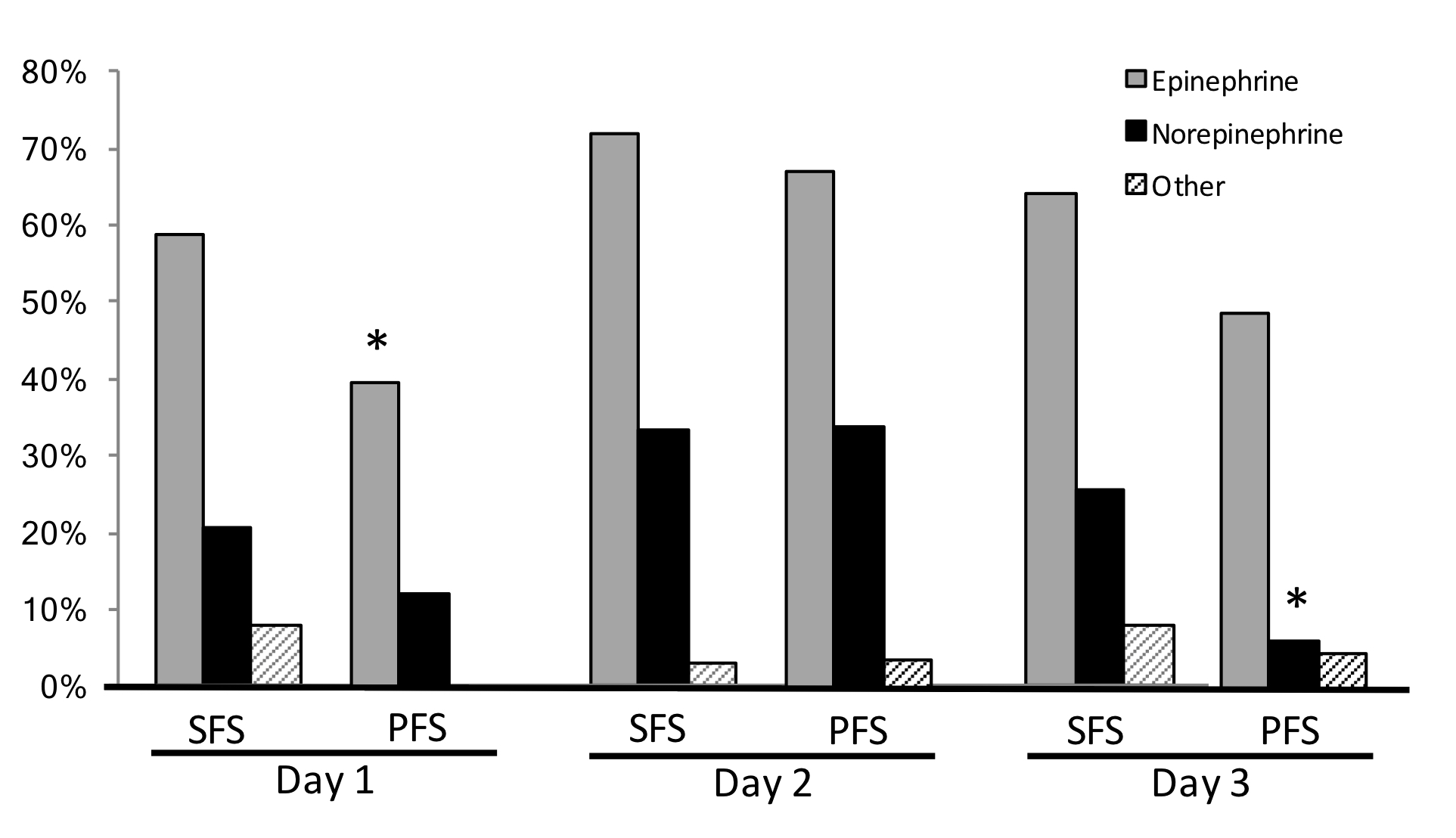

Supplement: Supplementary file 1 — Figure S1. Vasoactive drug use in standard fluid strategy and preemptive fluid strategy at day 1, 2 and 3 of study. * P < 0.05. Abbreviations: PFS: preemptive fluid strategy; SFS: standard fluid strategy. (JPG 226 kb) [file 12887_2018_1188_MOESM1_ESM.jpg]
